# Olfactory Stimulation Enhances Trigeminal Responses at the Mucosal Level

**DOI:** 10.1002/lary.32173

**Published:** 2025-04-07

**Authors:** Yiling Mai, Georg Karl Ludwig Burghardt, Thomas Hummel

**Affiliations:** ^1^ Smell and Taste Clinic, Department of Otorhinolaryngology Technische Universität Dresden Dresden Germany

**Keywords:** interaction, negative mucosa potential (NMP), olfactory, peripheral, trigeminal

## Abstract

(1) Trigeminal stimulation induced a clear NMP; trigeminal‐olfactory co‐stimulation induced an even greater NMP. (2) Olfactory stimulation may enhance peripheral neural processing of trigeminal stimulation. (3) Olfactory‐trigeminal interaction might occur before reaching the central nervous system.

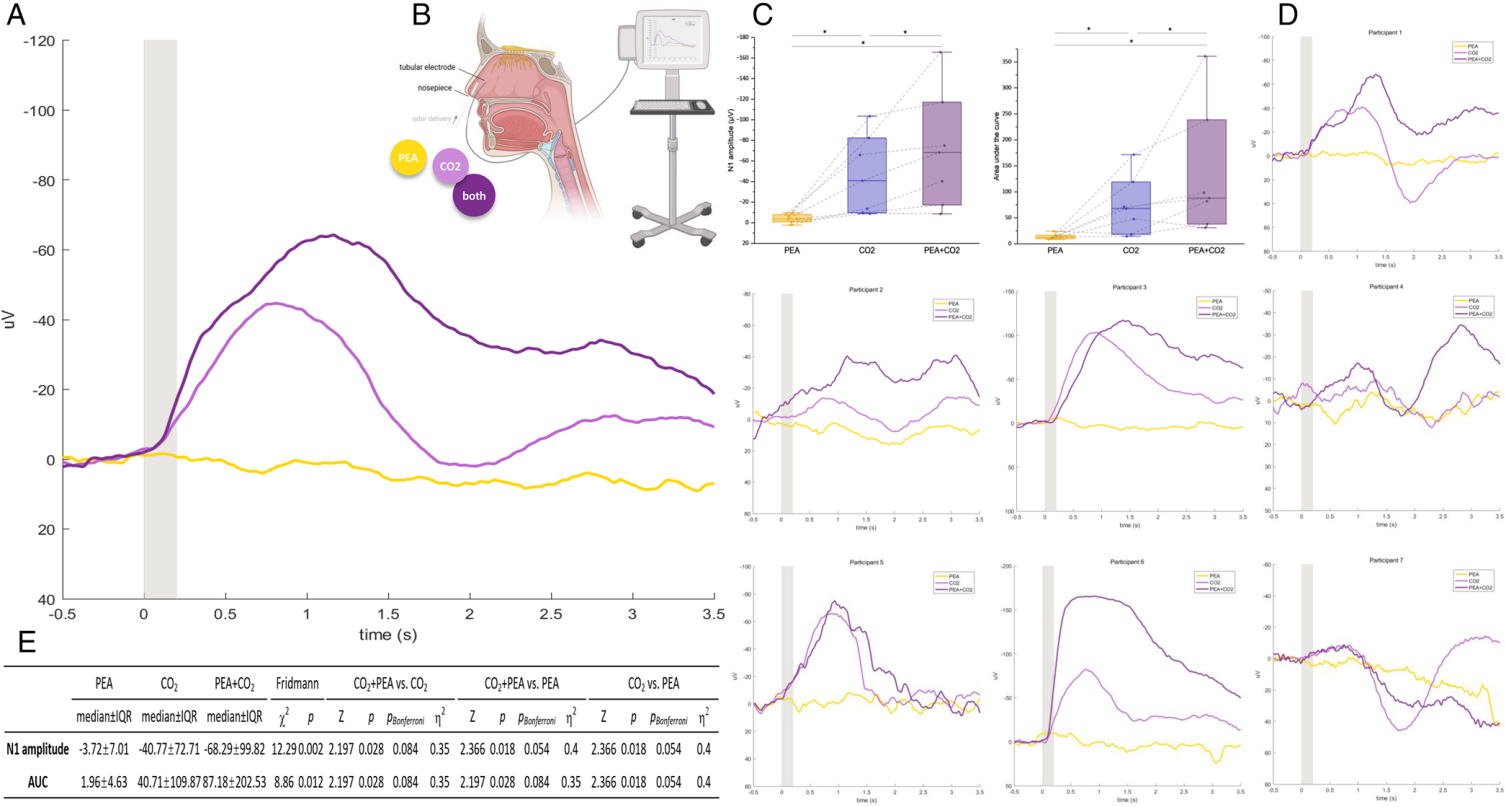

## 1 Introduction

The olfactory and trigeminal systems together shape the sensory experiences of odors [1]. The olfactory system detects odor molecules, while the trigeminal system mediates somatosensations such as irritation [1]. These two systems interact in ways that enhance or suppress each other [2]. Studies suggest that trigeminal stimulation often masks olfactory perception [3], which is related to the distorted peripheral responses of olfactory receptors due to trigeminal co‐stimulation [3] and possibly involves interactions at the central level [4]. In contrast, simultaneous olfactory stimulation enhances trigeminal sensations [2]. For example, co‐stimulation with strawberry and l‐isopulegol increased perceived intensity compared to either stimulus alone [5]. Such enhancement may occur centrally, as olfactory‐trigeminal stimulation elicited larger ERP amplitudes than either stimulus alone [5, 6] and that stimulating one nasal cavity with a trigeminal stimulus and simultaneously stimulating the contralateral side with an odorant amplifies the trigeminal percept similarly to simultaneous ipsilateral stimulation [7]. Peripheral mechanisms may also contribute, as behavioral studies suggest that ipsilateral but not contralateral olfactory‐trigeminal co‐stimulation improves trigeminal stimulus localization [8]. However, direct evidence on whether olfactory stimulation enhances trigeminal neural processing peripherally remains lacking. To address this gap, we utilized negative mucosa potential (NMP) recordings, which are electrophysiological representations of trigeminal activity from the respiratory mucosa where trigeminal nerves are distributed [9, 10, 11]. By delivering olfactory, trigeminal, and combined stimuli and recording NMP responses, this study examined whether peripheral trigeminal neural responses to olfactory‐trigeminal mixtures are enhanced compared to trigeminal stimulation alone.

**FIGURE 1:**
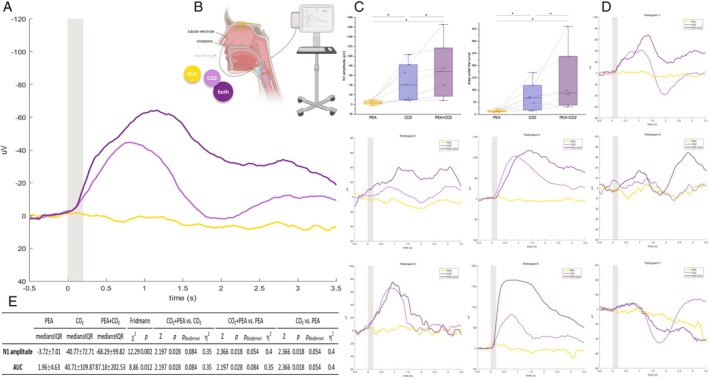
Negative mucosa potentials of olfactory, trigeminal, and their mixed stimulations. *Note*: Panel (A) shows the group‐level average negative mucosal potentials (NMP) over time (seconds) following stimulus onset, with the *y*‐axis representing response amplitude (μV). The shaded gray area indicates odor delivery. Panel (B) shows the placement of an Ag‐AgCl electrode on the anterior nasal septum under endoscopic control, stabilized with a clip mounted on a frame similar to lensless glasses for NMP recording. During the recording, participants received 20 CO_2_ (40% v/v), 20 phenylethyl alcohol (PEA, 40% v/v), and 20 CO_2_ + PEA (40% v/v each) stimuli in a pseudo‐randomized block order through a computer‐controlled olfactometer (constant airflow 6 L/min, temperature 36°C). Each stimulus lasted 200 ms with an inter‐stimulus interval of 18–20s. NMP data were processed using Letswave 7.0 (sampling rate: 120 Hz; bandpass: 0.01‐15 Hz). Panel (C) displays NMP N1 amplitudes and area under the curve (AUC) for each condition, showing median (line in the box), interquartile range (IQR, upper and lower limits of the box), max‐min range (whiskers), and individual data points connected across conditions with dashed lines. Significant post hoc pairwise comparisons without Bonferroni correction are indicated by an asterisk (*p < 0.05), as Bonferroni correction can be overly conservative for small sample sizes. However, to provide a comprehensive view, panel (E) also presents the p‐values with Bonferroni correction. Panel (D) shows individual averaged NMPs over time, similar to panel (A). Panel (E) shows the median ± IQR for each condition, along with the statistical group comparison (Friedman test) and corresponding post hoc pairwise comparison (Wilcoxon signed‐rank test) results. [Color figure can be viewed in the online issue, which is available at www.laryngoscope.com.]

Examination included seven healthy adults (38.7 ± 26.6 years, 4 women) with self‐reported normal olfaction and no nasal pathology, confirmed by ENT examination. NMP were recorded using an Ag‐AgCl electrode placed on the anterior nasal septum under endoscopic control (Figure 1). Participants received 20 stimuli each of CO_2_, phenylethyl alcohol (PEA), and CO_2_ + PEA stimuli through a computer‐controlled olfactometer. The maximum negative amplitude (N1) and area under the curve (AUC) were calculated to quantify the NMP.

Visual inspection showed CO_2_ produced clear NMPs, PEA induced barely detectable responses, while CO_2_ + PEA elicited NMPs greater than CO_2_ alone. This pattern was observed in six out of seven participants. The Friedman test revealed a significant difference in N1 amplitude and AUC across three conditions (*n* = 7; PEA vs. CO_2_ vs. CO_2_ + PEA [Median ± IQR]: N1 amplitude = −3.72 ± 7.01 vs. −40.77 ± 72.71 vs. −68.29 ± 99.82, *χ* ^2^ = 12.29, *p* = 0.002; AUC = 1.96 ± 4.63 vs. 40.71 ± 109.87 vs. 87.18 ± 202.53, *χ* ^2^ = 8.86, *p* = 0.012). Post hoc Wilcoxon signed‐rank test showed that both N1 amplitude and AUC for CO_2_ + PEA were greater than for CO_2_ alone (*Z* = 2.20, *p* = 0.028, *η* ^2^ = 0.35). Additional statistical details are provided in Figure 1.

## 2 Discussion

Via visual inspection, six out of seven participants showed a consistent pattern: olfactory stimulation induced minimal NMPs, trigeminal stimulation produced clear NMPs, and co‐stimulation elicited greater NMPs than trigeminal stimulation alone. Statistically, CO_2_ + PEA responses were greater than CO_2_ alone without strict p‐value adjustment, with a relatively large effect size of 0.35 [12]. This preliminary finding potentially implies that olfactory stimulation may modulate trigeminal processing peripherally, before reaching the central nervous system (CNS). Notably, given the small sample size of seven and the lack of statistical significance after stricter correction (i.e., Bonferroni), these observations require validation in larger samples.

Nevertheless, this initial observation raises the possibility of further investigating several speculations about how olfactory stimulation influences trigeminal activity in the respiratory epithelium, where olfactory nerves are absent: (1) PEA, though widely considered a “pure” olfactory stimulus, may induce subliminal trigeminal activation under certain conditions, like high volume/concentration [13]. If true, the greater PEA+CO_2_ NMPs might reflect “trigeminal‐trigeminal synergistic effect” rather than “olfactory‐trigeminal interaction.” However, this seems less likely in this study, because (a) a relatively low concentration (40%v/v) was used; (b) NMPs are highly sensitive to trigeminal activation and can be detected at “pre‐pain” sensations [14, 15], yet PEA NMPs were barely, if at all, detectable; and (c) a study [16] found that while healthy participants can localize PEA, anosmic patients cannot, suggesting its classification as a selectively olfactory stimulant in practice. While these points seem to favor the olfactory‐trigeminal interaction interpretation, replicating the present experiments in anosmic individuals is necessary to confirm the findings. (2) Given that olfactory structures were absent at the recording site, the observed peripheral interaction might be influenced by top‐down CNS feedback, as olfactory structures were absent at the recording site. Simultaneous ERP (scalp) and NMP recordings could help clarify the source of this interaction. (3) Unknown peripheral mechanisms, potentially involving chemical mediators or local neural circuits, might contribute, yet require further investigation with larger samples. From a practical perspective, this preliminary finding also implies that olfactory properties should be considered when analyzing trigeminal sensory encoding of mixed odors, as well as the multisensory integrative ability when assessing olfactory and trigeminal dysfunction in clinical contexts.

## 3 Conclusion

Olfactory stimulation might enhance peripheral trigeminal neural processing. This might reflect the multisensory integration of olfactory and trigeminal inputs, which potentially plays a role in amplifying environmental signals. However, these findings are preliminary. Studies with larger sample sizes and pathological cases are necessary to validate this initial observation.

## Ethics Statement

The study was performed at the Smell and Taste Clinic of the TU‐Dresden and conducted according to the Declaration of Helsinki and approved by the Ethics Committee at the Medical Faculty of the TU Dresden (application number: EK237072018). All participants provided informed written consent prior to the study.

## Conflicts of Interest

The authors declare no conflicts of interest.

## References

[1] T. Hummel and J. Frasnelli , “The Intranasal Trigeminal System,” Handbook of Clinical Neurology 164 (2019): 119–134, 10.1016/B978-0-444-63855-7.00008-3. 31604542

[2] G. Brand , “Olfactory/Trigeminal Interactions in Nasal Chemoreception,” Neuroscience and Biobehavioral Reviews 30, no. 7 (2006): 908–917, 10.1016/J.NEUBIOREV.2006.01.002. 16545453

[3] F. Genovese , J. Xu , M. Tizzano , and J. Reisert , “Quantifying Peripheral Modulation of Olfaction by Trigeminal Agonists,” Journal of Neuroscience 43, no. 47 (2023): 7958–7966, 10.1523/JNEUROSCI.0489-23.2023. 37813571 PMC10669757

[4] F. S. Müschenich , T. Sichtermann , M. E. Di Francesco , et al., “Some Like It, Some Do Not: Behavioral Responses and Central Processing of Olfactory–Trigeminal Mixture Perception,” Brain Structure & Function 226, no. 1 (2021): 247–261, 10.1007/S00429-020-02178-4. 33355693 PMC7817597

[5] A. Oleszkiewicz , R. Pellegrino , C. Guducu , L. Farschi , J. Warr , and T. Hummel , “Temporal Encoding During Unimodal and Bimodal Odor Processing in the Human Brain,” Chemosensory Perception 12, no. 1 (2019): 59–66, 10.1007/S12078-018-9251-0.

[6] A. Livermore , T. Hummel , and G. Kobal , “Chemosensory Event‐Related Potentials in the Investigation of Interactions Between the Olfactory and the Somatosensory (Trigeminal) Systems,” Electroencephalography and Clinical Neurophysiology 83, no. 3 (1992): 201–210, 10.1016/0013-4694(92)90145-8. 1381671

[7] W. S. Cain and C. L. Murphy , “Interaction Between Chemoreceptive Modalities of Odour and Irritation,” Nature 284, no. 5753 (1980): 255–257, 10.1038/284255a0. 7360255

[8] C. Tremblay and J. Frasnelli , “Olfactory and Trigeminal Systems Interact in the Periphery,” Chemical Senses 43, no. 8 (2018): 611–616, 10.1093/CHEMSE/BJY049. 30052799

[9] T. Hummel , H. G. Kraetsch , E. Pauli , and G. Kobal , “Responses to Nasal Irritation Obtained From the Human Nasal Mucosa,” Rhinology 36, no. 4 (1998): 168–172. 9923059

[10] N. Thürauf , M. Günther , E. Pauli , and G. Kobal , “Sensitivity of the Negative Mucosal Potential to the Trigeminal Target Stimulus CO_2_ ,” Brain Research 942, no. 1–2 (2002): 79–86, 10.1016/S0006-8993(02)02697-5. 12031855

[11] T. Meusel , S. Negoias , M. Scheibe , and T. Hummel , “Topographical Differences in Distribution and Responsiveness of Trigeminal Sensitivity Within the Human Nasal Mucosa,” Pain 151, no. 2 (2010): 516–521, 10.1016/J.PAIN.2010.08.013. 20817356

[12] W. Lenhard and A. Lenhard , “Computation of Effect Sizes,” https://www.psychometrica.de/effect_size.html. Psychometrica, 10.13140/RG.2.2.17823.92329.

[13] J. Frasnelli , T. Hummel , J. Berg , G. Huang , and R. L. Doty , “Intranasal Localizability of Odorants: Influence of Stimulus Volume,” Chemical Senses 36, no. 4 (2011): 405–410, 10.1093/CHEMSE/BJR001. 21310764 PMC3105605

[14] J. Frasnelli and T. Hummel , “Age‐Related Decline of Intranasal Trigeminal Sensitivity: Is It a Peripheral Event?,” Brain Research 987, no. 2 (2003): 201–206, 10.1016/S0006-8993(03)03336-5. 14499964

[15] J. A. Boyle , J. N. Londström , M. Knecht , M. Jones‐Gotman , B. Schaal , and T. Hummel , “On the Trigeminal Percept of Androstenone and Its Implications on the Rate of Specific Anosmia,” Journal of Neurobiology 66, no. 13 (2006): 1501–1510, 10.1002/NEU.20294. 17013929

[16] J. Porter , T. Anand , B. Johnson , R. M. Khan , and N. Sobel , “Brain Mechanisms for Extracting Spatial Information From Smell,” Neuron 47, no. 4 (2005): 581–592, 10.1016/J.NEURON.2005.06.028. 16102540

